# Articaine in dentistry: an overview of the evidence and meta-analysis of the latest randomised controlled trials on articaine safety and efficacy compared to lidocaine for routine dental treatment

**DOI:** 10.1038/s41405-021-00082-5

**Published:** 2021-07-17

**Authors:** Erica Martin, Alan Nimmo, Andrew Lee, Ernest Jennings

**Affiliations:** 1 General Dentistry, College of Medicine and Dentistry, James Cook University, Cairns, QLD Australia; 2 Medical Science, College of Medicine and Dentistry, James Cook University, Cairns, QLD Australia; 3 Preventative Dentistry and Indigenous Oral Health, College of Medicine and Dentistry, James Cook University, Cairns, QLD Australia; 4 Anatomy, College of Medicine and Dentistry, James Cook University, Cairns, QLD Australia

**Keywords:** Dental anaesthesia, Dentistry

## Abstract

**Objectives:**

To comprehensively review the existing studies of articaine in dentistry and conduct a systematic review and meta-analysis to answer the following Population, Intervention, Comparison and Outcome question: “Is articaine a safe and efficacious local anaesthetic for routine dental treatment compared to lidocaine?”

**Methods:**

Database searches were conducted in Medline Ovid, Medline Pubmed, Scopus, Emcare, Proquest and the Cochrane Central register of Controlled Trials. Inclusion criteria were all existing English, human, randomised controlled trials of interventions involving 4% articaine and 2% lidocaine in routine dental treatment. Twelve studies were included for meta-analysis using Cochrane Review Manager 5 software. Anaesthetic success odds ratios were calculated using a random-effects model.

**Results:**

Articaine had a higher likelihood of achieving anaesthetic success than lidocaine overall and in all subgroup analyses with varying degrees of significance. Overall (OR: 2.17, 95% CI: 1.50, 3.15, *I*^2^ = 62%) articaine had 2.17 times the likelihood of anaesthetic success of lidocaine (*P* < 0.0001). For mandibular blocks (OR: 1.50, 95% CI: 1.14, 1.98, *I*^2^ = 0%) articaine had 1.5 times the likelihood of anaesthetic success of lidocaine (*P* = 0.004). For all infiltrations, maxillary and mandibular (OR: 2.78, 95% CI: 1.61, 4.79, *I*^2^ = 66%) articaine had 2.78 times the likelihood of anaesthetic success of lidocaine (*P* = 0.0002). None of the studies reported any major local anaesthetic-related adverse effects as a result of the interventions.

**Conclusions:**

Articaine is a safe and efficacious local anaesthetic for all routine dental procedures in patients of all ages, and more likely to achieve successful anaesthesia than lidocaine in routine dental treatment. Neither anaesthetic has a higher association with anaesthetic-related adverse effects.

## Introduction

Local anaesthetics (LAs) provide pain-free patient dental care reducing patient anxiety and phobia. Evidence-based dental clinical practice should be based upon the latest clinical research with continuous re-assessment of all available clinical data on dental anaesthetic efficacy and safety.

### Purpose of this review

The aim of this research is twofold: to review the existing studies of articaine use for routine dental treatment and to conduct a meta-analysis of randomised control trials answering the following Population, Intervention, Comparison and Outcome question: Is articaine as safe and efficacious as the current gold standard dental anaesthetic, lidocaine for all routine dental treatment? For the purposes of this review, the definition of routine dental treatment are standard dental procedures taught in mainstream undergraduate dental curriculums.

Systematic reviews are considered the most robust method for summarizing large volumes of study evidence, and meta-analyses of research data are considered the highest form of evidence.^1,2^ The Cochrane Handbook for Systematic Reviews of Interventions recommends that review data should be updated every 2 years or when relevant new data emerges in the literature.^3^

The research questions for this research paper are: “Is articaine a safe LA for all routine dental treatment?” and “Is articaine as safe and efficacious as the current gold standard dental anaesthetic, lidocaine for all routine dental treatment?”

### Articaine pharmacology

Articaine, 4-methyl-3[2-(propylamino)-propionamido]-2-thiophene-carboxylic acid, methyl ester hydrochloride, belongs to the amide family of LAs, which also includes lidocaine, mepivacaine, bupivicaine and prilocaine.^4–6^ Articaine is unique amongst the amide family, containing an ester group and having a thiophene instead of a benzene ring.^4–6^ The thiophene ring, an integral feature of articaine’s LA potency^7^ increases articaine’s lipid solubility facilitating more efficient diffusion of the anaesthetic through the nerve cell lipid membrane and into surrounding tissue.^8–10^ A 2000 pharmacological study of various anaesthetic diffusion across nerve membranes found that articaine’s lipid-soluble abilities result in superior diffusive action of articaine when compared with other LA formulas.^7^

Articaine has a serum half-life of 20–30 min, shorter than the other amide LAs due to the more rapid hydrolysis of the ester group within the plasma.^5,9,11,12^ Lidocaine has a half-life of 90–120 min.^9^ Articaine’s ester group allows 90%^5,11^ of the anaesthetic to metabolise within the plasma to the inert metabolite, articainic acid, and be excreted via the kidneys resulting in the shorter half-life compared to the other amide LAs. The remaining 10% biotransforms within the liver.^12^

Oertel^5^ concluded that articaine’s shorter half-life means that articaine can be given safely at higher concentrations;^5^ however, Paxton and Thorne^8^ argue that lipid solubility may not determine the speed of diffusion across the cell membrane.^8^ Other studies have proposed that anaesthetic binding to plasma proteins has greater association with ionic channel action than lipid solubility.^5^ Similar to the other amide LAs, articaine anaesthetises tissue by blocking nerve conduction. The addition of a vasoconstrictor prolongs the anaesthetic effect by delaying absorption of the anaesthetic solution.^12^

Studies investigating the pharmacology and toxicology of articaine in animals recognised that articaine had 1.5 times higher anaesthetic efficiency, superior ability in infiltration anaesthesia and low toxicity to local tissues when compared with the other amide LAs.^8^ A rat sensory nerve conduction study concluded that 2% and 4% articaine more effectively anaesthetise nerve fibres than other LAs.^13^ Articaine’s anaesthetic effect lasts ~120 min, which is similar to lidocaine.^5^

### Articaine in dentistry

Articaine was first synthesized in Germany in 1969 under the label, HOE 40-045, and then released for clinical use in 1976 under the name, Carticaine hydrochloride.^6,9^ Winther and Nathalang conducted the first clinical trials of articaine in 1971 finding that 2% articaine with 1:200,000 adrenaline was superior to 2% lidocaine with 1:200,000 adrenaline in anaesthetic duration and extent, and that articaine produced profound anaesthesia for all teeth except mandibular molars.^4^ In 1984, carticaine was renamed to articaine^8^ and in 2000, was approved by the US FDA as a 4% formula with 1:100,000 adrenaline under the name Septocaine (Septodont). The FDA approved 4% articaine with 1:200,00 adrenaline in 2006.^6^

### Articaine efficacy

Articaine LA onset takes between 1.5 and 1.8 min for a maxillary infiltration and 1.5–3.6 min for mandibular block anaesthesia.^4,6,14^ Articaine pulpal anaesthesia lasts between 30 and 120 min, a duration longer than lidocaine, mepivacaine and prilocaine.^4^ Articaine soft tissue anaesthesia lasts ~2.25 h for maxillary infiltrations and 4 h for mandibular blocks.^6^

### Articaine safety

Malamed et al.’s 2001’s multi-centre trial involving the comparison of 2% lidocaine with 4% articaine on 1325 patients aged 4–80 years of age, found that articaine was well-tolerated and safe for use in routine clinical dentistry.^6^ Both anaesthetics are appropriate and effective for clinical use. Articaine’s toxicity is comparable to that of lidocaine,^4,12^ but Malamed et al. cautioned use of both lidocaine and articaine in patients with liver or cardiovascular impairment as amide biotransformation occurs in the liver and the anaesthetics can decrease myocardial function for patients with advanced cardiovascular disease.^6^

### Lidocaine and articaine use in dentistry

Lidocaine has proven safe and efficacious for routine clinical treatment.^9^ Lidocaine entered the clinical market in 1948 and has since been the most common dental LA in most countries.^8^ Lidocaine sets the dental LA gold standard against which all new LAs are compared.^9^

Despite the popularity of lidocaine, dental LA reviews in 1995,^15^ and 2000^16^ recognised articaine’s growing popularity stating that articaine was the most popular dental anaesthetic in some countries at the time. Oertel’s^5^ review of articaine stated that lidocaine was the LA most used in dentistry, but that articaine was well-established as a mainstream dental LA in continental Europe and Canada, and the most widely used dental LA in Germany, Italy and the Netherlands.^5^ A 1989 study of German dentists found that articaine is used 72% of the time and lidocaine 13% of the time.^17^ A 2005 study by Vree and Gielen stated that ‘in dentistry, articaine is the drug of choice in the vast majority of the literature’.^18^

## Material and methods

Population, Intervention, Comparison and Outcome question: Is articaine as safe and efficacious as the current gold standard dental anaesthetic, lidocaine for all routine dental treatment?”

– Population: routine dental treatment
– Intervention: 4% articaine dental local anaesthesia
– Comparison: 2% lidocaine dental local anaesthesia
– Outcome: dental local anaesthesia efficacy and safety

The systematic review was registered in the PROSPERO database prior to the literature search.^19^ The search strategy follows the PRISMA-preferred reporting items for systematic reviews and meta-analysis.^20^

### Search terms

MeSH terms search: Exp dental anaesthetic, Exp articaine, Exp randomized controlled trial

Text word search: ‘local an?aesthetic’ OR ‘dental an?aesthetic’; carticaine OR articaine OR septanest OR septocaine OR ultracaine; (randomized controlled trial OR clinical trial OR exp clinical trial OR random* OR trial? OR review)

Databases searched: Medline Ovid, Medline Pubmed, SCOPUS, Cochrane Central Register of Controlled Trials, Emcare Ovid, ProQuest

Ongoing articaine trials were reviewed for redundancy on the PROSPERO International prospective register of systematic reviews.

### Selection of studies

Inclusion criteria for the search:

– All existing online studies of interventions involving articaine from its release to February 2020
– Randomised controlled trials (RCTs)
– Studies of routine dental procedures
– Studies published in English

The outcomes measures for the systematic review included: anaesthetic success, anaesthetic onset and duration and post intervention LA-related adverse events.

The initial search of the listed databases resulted in 1449 studies.

### Search methodology

From the initial 1449 results, a subsequent title and abstract review excluded 617 duplicates and 832 studies based upon the following exclusion criteria:

– Non-English studies
– Trials on non-humans
– Complex dental procedures involving soft tissue surgery and bone removal
– Medically compromised patients
– Digital anaesthesia and non-routine dental anaesthetic techniques e.g. intraosseous, intraligamentary, intra-pulpal, intra-pocket anaesthesia, non-standard mandibular block techniques (Gow-Gates and Vazarani–Akinosi techniques)

– Unrecognised duplicates
– Interventions not including lidocaine or articaine
– Full text not available

**Fig. 1:**
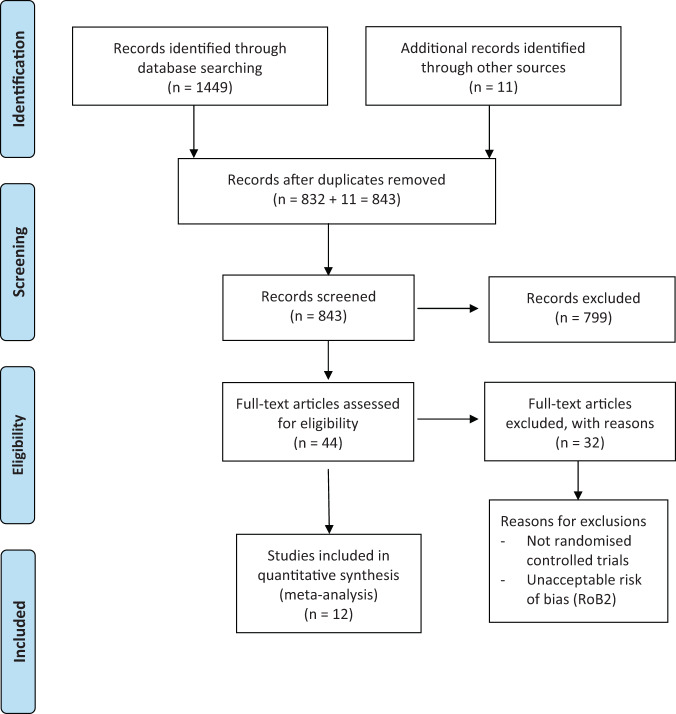
PRISMA flowchart of the search process.

A full text review was conducted on 42 studies, of which, nine were further excluded for being incomplete or not RCTs. A review of citations from previous systematic reviews of articaine and the included studies revealed 11 more sources. A search of the grey literature databases did not produce any further sources. The final search resulted in 44 randomised controlled studies comparing 4% articaine to 2% lidocaine (Fig. 1).

### Risk of bias assessment

Forty-four RCTs were reviewed by the researcher for risk of bias according to Cochrane Risk of Bias 2 guidelines.^21^

Cochrane Risk of Bias 2 guidelines include assessments of bias from:

– The randomization process (allocation sequence and concealment)
– Deviations from intended interventions (extent/quality of blinding and balanced interventions)
– Missing outcome data
– Measurement of the outcome (quality and appropriateness)
– Selection of the reported result

The risk of bias was assessed as: low, high, unknown risk or some concerns. Low-risk studies had no concerns judged in any domains. Any study with a single concern was judged as ‘some concerns’. Studies with multiple concerns or deemed high risk in any domain was judged as ‘high risk’ and studies with no information were deemed ‘unknown risk’. Studies with multiple concerns or any high-risk category were excluded from the meta-analysis.

**Fig. 2:**
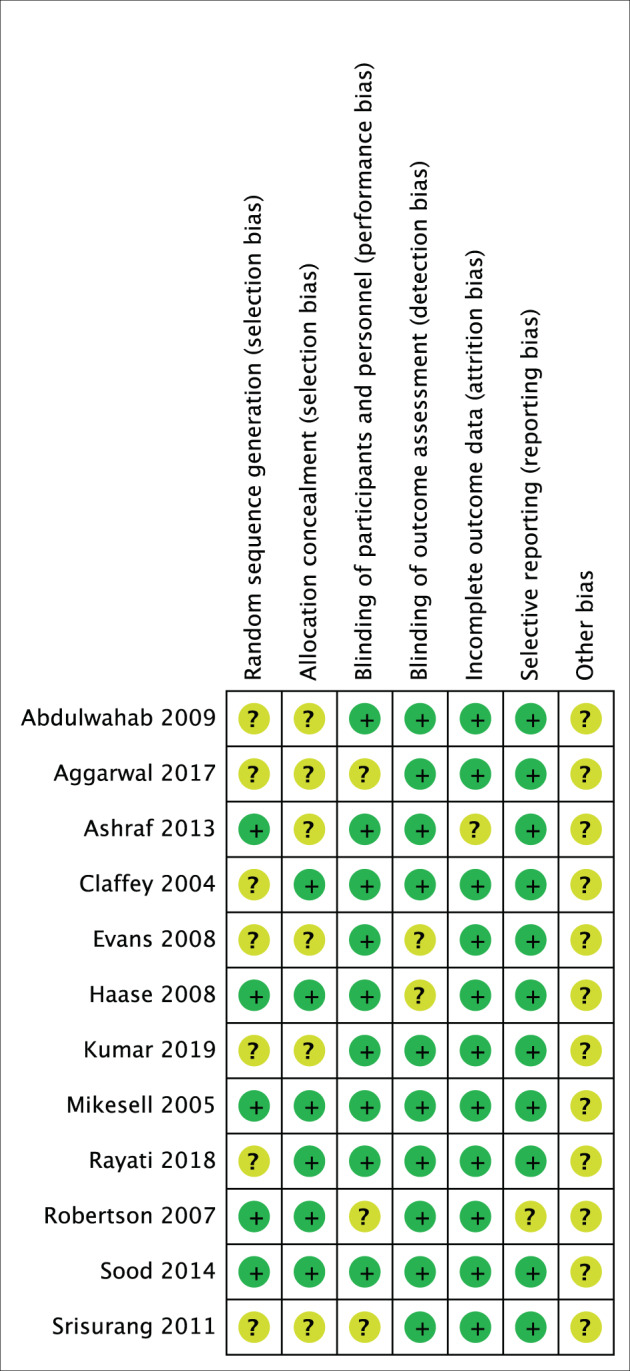
Risk of bias summary.

Thirteen studies were assessed as ‘low’ or ‘some concern’ risk of bias. One study from 1993 was not included for meta-analysis due to lack of appropriate study data measurements. Twelve studies were included in the meta-analysis (Fig. 2).

The process was assessed by another of the authors and any differences were resolved for final consensus by an independent third-party reviewer.

### Data extraction/study characteristics

Data from the final 12 studies were extracted onto a Microsoft EXCEL spreadsheet (Table 1).

**Table 1:** Characteristics of included studies.

Reference	Sample size	Age range (m)	Sex distribution	Arch	Teeth	Pre-operative tooth status	Anaesthetic technique	Anaesthetic dose	Vasoconstrictor concentration	Assessment method	Anaesthetic success %	Adverse effects
Abdulwahab et al.^39^	18	18–53 *m* = 24.9	M6 F12	Md	Molar	Healthy asymptomatic	BI	0.9 mLs	A 1:100,000 L 1:100,000	EPT	A38.9 L16.7	Minor/no reported paraesthesia
Aggarwal et al.^33^	91	24–47 *m* = 34	M57 F34	Md	Molar	Irreversible pulpitis	IANB	1.8 mLs	A 1:100,000 L 1:200,000	Endo access	A33 L23	No data
Ashraf et al.^35^	102	20–60	M47 F55	Md	Molar	Irreversible pulpitis	BI following failed IANB	1.5 mLs	A 1:100,000 L 1:100,000	Endo access	A71 L29	No data
Claffey et al.^30^	72	20–53 *m* = 31	M25 F47	Md	Molar or premolar	Irreversible pulpitis	IANB	2.2 mLs	A 1:100,000 L 1:100,000	Endo access	A24 L23	No data
Evans et al.^36^	80	20–36 *m* = 25	M46 F34	Mx	Lat. Incisor/molar	Healthy asymptomatic	Infiltration	1.8 mLs	A 1:100,000 L 1:100,000	EPT	LI A88 L62/Molar A78 L72	Minor/no reported paraesthesia
Haase et al.^34^	73	20–36 *m* = 27	M46 F27	Md	Molar	Healthy asymptomatic	BI following failed IANB	1.8 mLs	A 1:100,000 L 1:100,000	EPT	A88 L71	Minor/no reported paraesthesia
Kumar et al.^40^	100	20–59 *m* = 27	M54 F46	Mx	Molar	Needing extraction	Infiltration	1.8 mLs	A 1:100,000 L 1:100,000	EPT	A44 L48	No adverse effects/no reported paraesthesia
Mikesell et al.^31^	57	*m* = 28	M30 F27	Md	Molars, premolars	Healthy asymptomatic	IANB	1.8 mLs	A 1:100,000 L 1:100,000	EPT	A48.6 L37.7	Minor/no reported paraesthesia
Rayati et al.^38^	133	20–50	M65 F68	Md	Molars	Needing extraction	BI	1.8 mLs	A 1:100,000 L 1:100,000	Extraction	A18 L1	No data
Robertson et al.^37^	60	19–51 *m* = 27	M26 F34	Md	Molar or premolar	Healthy asymptomatic	BI	1.8 mLs	A 1:100,000 L 1:100,000	EPT	A84.8 L57.3	Minor/no reported paraesthesia
Sood et al.^32^	100	18–50 *m* = 27	no data	Md	Molars	Irreversible pulpitis	IANB	1.8 mLs	A 1:100,000 L 1:80,000	Endo access	A88 L82	No data
Srisurang et al.^42^	33	13-45 *m* = 18.2	no data	Mx	Premolars	Needing extraction	Infiltrations	1.2 mLs	A 1:100,000 L 1:100,000	EPT	A100 L97	Minor/no reported paraesthesia

*Md* mandibular, *Mx* maxillary, *IANB* inferior alveolar nerve block, *BI* buccal infiltration, *EPT* electronic pulp tester.

### Data analysis

Data from 922 interventions were included in the meta-analyses.

Cochrane Review Manager 5.3 software (RevMan Version 5.3, The Nordic Cochrane Centre, The Cochrane Collaboration, Copenhagen, Denmark) was used to statistically analyse the principal outcome—anaesthetic success.

Analysis was performed for:

– All interventions in the studies—maxillary and mandibular infiltrations, and mandibular blocks*
– All mandibular interventions—block and infiltration studies
– Only mandibular block studies
– Only mandibular infiltration studies
– All infiltrations studies—maxillary and mandibular
– Only maxillary infiltration studies
– Pre-operative pulp status—asymptomatic versus symptomatic
– Study design—parallel versus crossover

*Mandibular block anaesthesia refers to inferior alveolar nerve blocks, as none of the included studies involved mental or incisive nerve blocks.

The principal summary measures were odd ratios calculated using a Mantel–Haenszel random-effects model for dichotomous data. Treatment differences between articaine and lidocaine were illustrated through forest plots.

Statistical heterogeneity was assessed using Tau^2^, Cochran Q test (Chi^2^) and the *I*^2^ test for inconsistency. Significance was set at *P* < = 0.05. Heterogeneity refers to variability in the intervention effects being evaluated and is a consequence of clinical or methodological diversity. Tau^2^ reflects the amount of variation found among the different studies in a random-effects model and reflects the amount of true heterogeneity. The Cochran Q-test assesses whether the true treatment effects are the same in all the primary studies and is expressed as a *P* value determining significant heterogeneity or not. *I*^2^ quantifies the statistical heterogeneity and represents the amount of variability in effect estimates.^22^

A sensitivity analysis of individual study effects on the pooled effects was assessed by omitting studies one by one and noting the change in overall odds ratio.

**Fig. 3:**
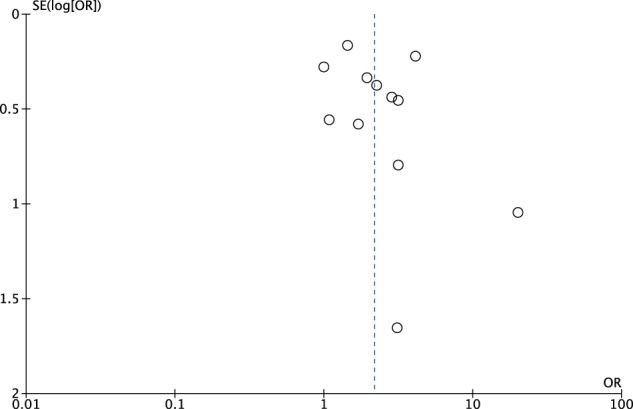
Funnel plot of all studies including outlier (Rayati et al.). All studies except one outlier fall within the funnel (shown in Fig. 4).

**Fig. 4:**
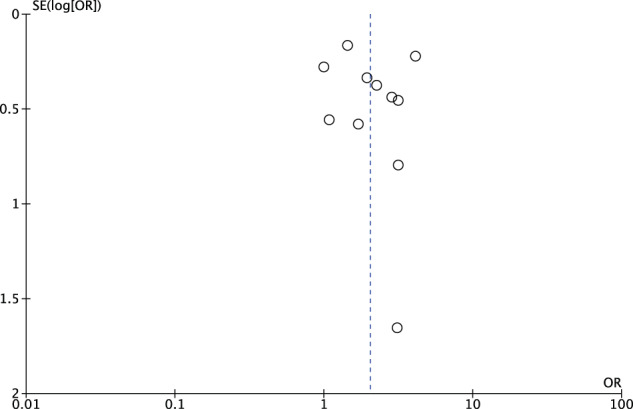
Funnel plot of studies minus outlier.

A funnel plot was used for assessment of publication bias (Figs. 3 and 4).

## Results

### Meta-analysis

The included studies showed medium-to-high levels of heterogeneity, therefore, a random-effects model and the Mantel–Haenszel statistical method was used for data analysis. Tooth and arch location, anaesthetic delivery method, anaesthetic volume, vasoconstrictor volume, pre-intervention tooth status and study type accounted for the variations between the studies.

In overall and subgroup analyses, articaine showed a higher likelihood of successful anaesthesia than lidocaine, with varying degrees of significance.

### Group analysis

**Fig. 5:**
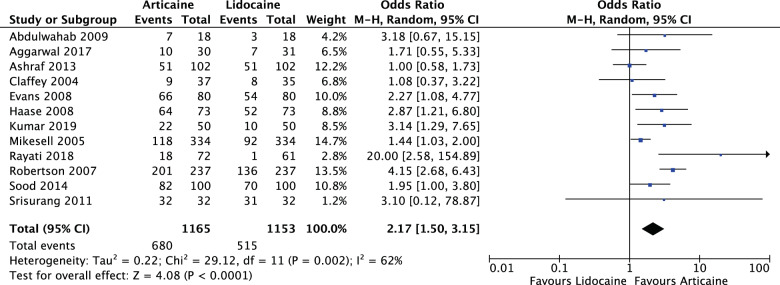
Forest plot—all local anaesthetic interventions. Articaine had 2.17 times the likelihood of anaesthetic success of lidocaine.

For all LA interventions (OR: 2.17, 95% CI: 1.50, 3.15, *I*^2^ = 62%), articaine had 2.17 times the likelihood of anaesthetic success of lidocaine. The results were significant (*P* < 0.0001) (Fig. 5).

### Subgroup analyses

#### Anaesthetic delivery method

**Fig. 6:**
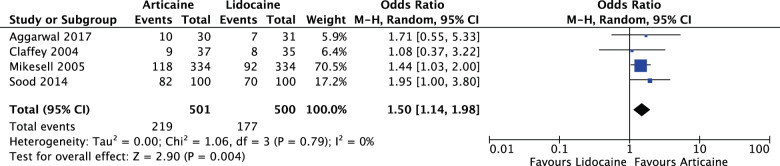
Forest plot—mandibular inferior alveolar nerve blocks. Articaine had 1.5 times the likelihood of anaesthetic success of lidocaine.

For mandibular blocks (OR: 1.50, 95% CI: 1.14, 1.98, *I*^2^ = 0%), articaine had 1.5 times the likelihood of anaesthetic success of lidocaine. The results were significant (*P* = 0.004) (Fig. 6).

**Fig. 7:**
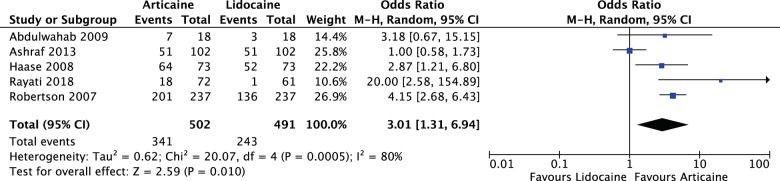
Forest plot—mandibular infiltrations. Articaine had 3.01 times likelihood of anaesthetic success of lidocaine.

For mandibular infiltrations (OR: 3.01, 95% CI: 1.31, 6.94, *I*^2^ = 80%), articaine had 3.01 times likelihood of anaesthetic success of lidocaine. The results were significant (*P* = 0.010) (Fig. 7).

**Fig. 8:**
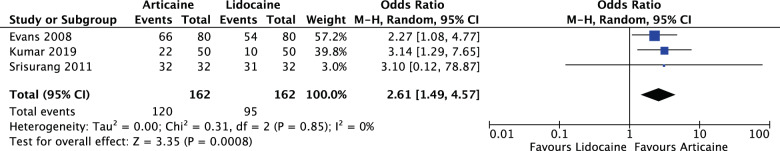
Forest plot—maxillary infiltrations. Articaine had 2.61 times likelihood of anaesthetic success of lidocaine.

For maxillary interventions (infiltrations) (OR: 2.61, 95% CI: 1.49, 4.57, *I*^2^ = 0%) articaine had 2.61 times likelihood of anaesthetic success of lidocaine. The results were significant (*P* = 0.0008) (Fig. 8).

**Fig. 9:**
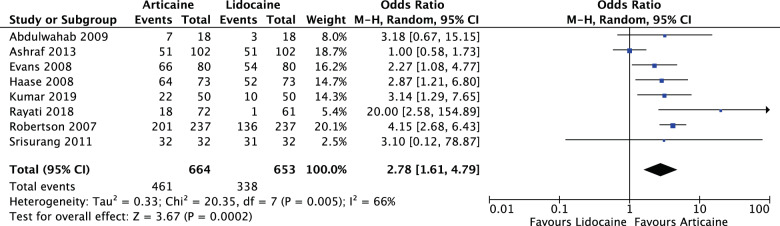
Forest plot—all infiltrations, maxillary and mandibular. Articaine had 2.78 times likelihood of anaesthetic success of lidocaine.

For all infiltrations, maxillary and mandibular (OR: 2.78, 95% CI: 1.61, 4.79, *I*^2^ = 66%), articaine had 2.78 times likelihood of anaesthetic success of lidocaine. The results were significant (*P* = 0.0002) (Fig. 9).

#### Arch difference

**Fig. 10:**
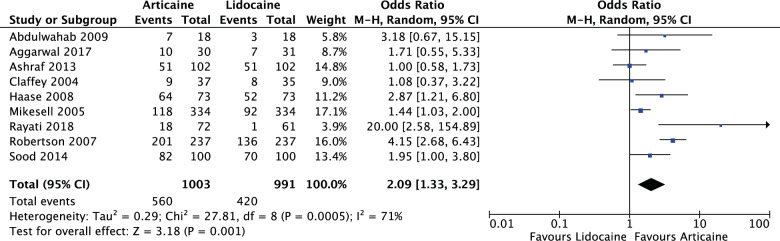
Forest plot—all mandibular interventions—blocks and infiltrations. Articaine had 2.09 times likelihood of anaesthetic success of lidocaine.

For all mandibular interventions (OR: 2.09, 95% CI: 1.33, 3.29, *I*^2^ = 71%), articaine had 2.09 times likelihood of anaesthetic success of lidocaine. The results were significant (*P* = 0.001) (Fig. 10).

For analysis of maxillary interventions (infiltrations) (OR: 2.61, 95% CI: 1.49, 4.57, *I*^2^ = 0%), articaine had 2.61 times likelihood of anaesthetic success of lidocaine. The results were significant (*P* = 0.0008) (Fig. 8).

#### Pre-intervention pulp status

**Fig. 11:**
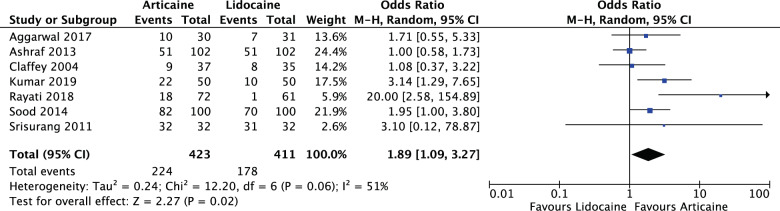
Forest plot—studies with pre-operative symptomatic teeth. Articaine had 1.89 times likelihood of anaesthetic success of lidocaine.

For all symptomatic teeth in the meta-analysis (OR: 1.89, 95% CI: 1.09, 3.27, *I*^2^ = 51%), articaine had 1.89 times likelihood of anaesthetic success of lidocaine. The results were significant (*P* = 0.02) (Fig. 11).

**Fig. 12:**
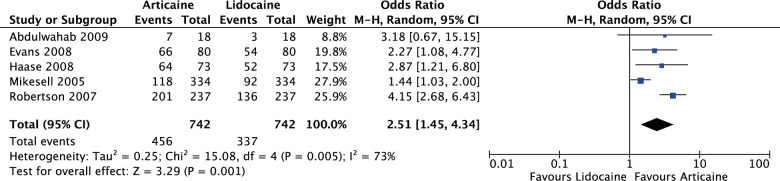
Forest plot—studies with pre-operative healthy teeth. Articaine had 2.51 times likelihood of anaesthetic success of lidocaine.

For all asymptomatic teeth in the meta-analysis (OR: 2.51, 95% CI: 1.47, 4.34, *I*^2^ = 73%), articaine had 2.51 times likelihood of anaesthetic success of lidocaine. The results were significant (*P* = 0.001) (Fig. 12).

#### Study design

**Fig. 13:**
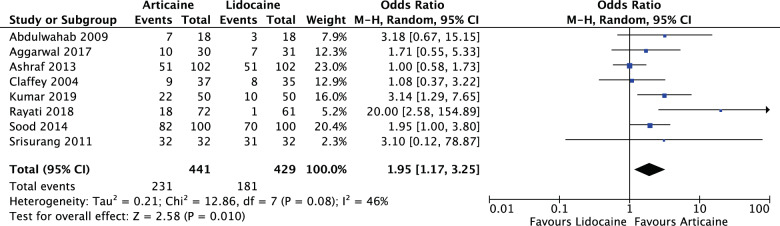
Forest plot—all parallel randomised controlled trial studies. Articaine had 1.95 times likelihood of anaesthetic success of lidocaine.

For all parallel studies (OR: 1.95, 95% CI: 1.17, 3.25, *I*^2^ = 46%), articaine had 1.95 times likelihood of anaesthetic success of lidocaine. The results were significant (*P* = 0.010) (Fig. 13).

**Fig. 14:**
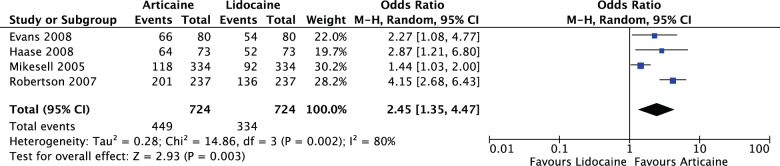
Forest plot—all crossover randomised controlled trial studies. Articaine had 2.45 times likelihood of anaesthetic success of lidocaine.

For all crossover studies (OR: 2.45, 95% CI: 1.35, 4.47, *I*^2^ = 80%), articaine had 2.45 times likelihood of anaesthetic success of lidocaine. The results were significant (*P* = 0.003) (Fig. 14).

### Publication bias funnel plot

A funnel plot was used to assess publication bias. Most studies fell within the funnel with one outlier, but the sensitivity effect was insignificant for omission any of the studies (Figs. 3 and 4).

### Adverse effects

Of the 12 included studies in this meta-analysis, four did not include data on LA-related adverse effects, the remaining nine stated that there were only minor temporary side-effects with no reported incidence of paraesthesia.

## Discussion

### Meta-analysis

The meta-analysis included data from human, randomised controlled trials based in U.S.A., India, Iran, Thailand and Finland, published in English between 1993 and 2019 involving intervention on 922 patients with asymptomatic or symptomatic pre-clinical tooth status and anesthetised with 4% articaine and 2% lidocaine anaesthetic for routine dental treatment. The studies included interventions on healthy teeth, teeth diagnosed with symptomatic irreversible pulpitis and teeth requiring extraction. The differences in pre-operative baseline pulp status were analysed for their effect in the meta-analysis because symptomatic teeth have been shown to be more difficult to anaesthetise than asymptomatic teeth.^11,23–26^

Data measurement tools in the studies included assessment of pulp status using electronic pulp testers, pain assessment using the 100 or 170-mm visual analogue scales, endodontic access success and extraction success. Anaesthetic success was the primary outcome measure for all the studies. Other outcome measures were assessment of pain during various stages of anaesthetic administration, pain during intervention, post-operative pain at 0, 24, 48 and 72 h, onset of pulpal anaesthesia, duration of pulpal anaesthesia and extent of soft tissue anaesthesia.

Electronic pulp testers have been the standard measurement tool used to ascertain pulpal status in quantitative clinical trials of dental anaesthetic setting the score of 80 as the criteria for complete pulpal anaesthesia.^27^ Symptomatic teeth may be more difficult to anaesthetise than asymptomatic teeth and pulpal anaesthesia of teeth with irreversible pulpitis is not guaranteed even with an electronic pulp tester score of 80 or more.^27^ Visual analogue scales of 100 and 170 mm were used in most of the included studies to quantify subjective pain data for valid analysis.^28,29^

This systematic review and meta-analysis recognise articaine as a safe and efficacious dental LA for all routine dental treatment. Compared to lidocaine, articaine is more efficacious in block and infiltration anaesthesia in both arches.

#### Mandibular block anaesthesia

In this review, mandibular block anaesthesia refers to the traditional inferior alveolar nerve block. Overall, articaine performed better than lidocaine in mandibular block anaesthesia for healthy and symptomatic teeth. Previously, most individual studies found that the differences were not statistically significant.^30–33^ Our meta-analysis found that, for mandibular block anaesthesia, articaine had 1.5 times the likelihood of anaesthetic success of lidocaine with statistical significance (*P* = 0.005). However, neither lidocaine nor articaine mandibular block anaesthesia adequately anaesthetised symptomatic teeth with irreversible pulpitis.^30,32,33^

#### Supplementary buccal infiltration following failed mandibular block anaesthesia

Our review corroborates previous review findings that articaine gives significantly more efficacious anaesthesia than lidocaine for supplementary buccal infiltration following failed mandibular block anaesthesia for healthy teeth and symptomatic teeth requiring endodontic treatment.^34,35^

#### Infiltrations

Articaine has a higher likelihood of anaesthesia success than lidocaine for: mandibular molar buccal infiltration anaesthesia,^36–38^ maxillary incisor infiltration anaesthesia^39^ and maxillary molar infiltration anaesthesia.^40^ A 1993 outlier study of maxillary anaesthesia noted no significant difference in LA success between articaine and lidocaine in terms of onset or duration.^41^ Our meta-analysis found that, for infiltrations, articaine had 2.78 times likelihood of anaesthetic success of lidocaine (*P* = 0.0002) and 3.01 times for mandibular infiltrations and 2.61 times for maxillary infiltrations (*P* = 0.01).

#### Arch

For both arches, the meta-analysis found that articaine had higher likelihood of anaesthesia success than lidocaine, 2.76 times more likely in the mandible (*P* = 0.0002) and 2.61 times more likely in the maxilla (*P* = 0.0008).

#### Pulp status

Meta-analysis was performed for the differences in pre-intervention pulp status between symptomatic and asymptomatic teeth. For asymptomatic teeth, articaine had 2.31 times higher likelihood of anaesthesia success of lidocaine with significance (*P* = 0.006). For symptomatic teeth, articaine had 1.89 times higher likelihood of anaesthesia success of lidocaine with significance (*P* = 0.02).

#### Study design

Meta-analysis was performed for the differences in parallel compared to crossover studies. Study design appeared not to influence anaesthesia outcomes in this meta-analysis. The included parallel and crossover RCTs showed that articaine had a higher likelihood of anaesthesia than lidocaine.

#### Extractions

Articaine can be used with buccal infiltration anaesthesia for successful extraction of maxillary premolars^42^ and maxillary molars without the need for palatal infiltrations,^40^ but should not replace standard mandibular block anaesthesia for extraction of mandibular molars.^38^

#### Anaesthetic onset and duration

All relevant studies showed faster onset and longer duration for articaine anaesthesia over lidocaine with varying degrees of significance. One study in the meta-analysis recorded data on anaesthetic onset, finding that the onset time for articaine mandibular buccal infiltration anaesthesia ranged from 4.2 to 4.7 min compared with 6.1 to 11.1 min for lidocaine.^37^ Two studies documented anaesthetic duration, one for maxillary molars infiltrations, with ~71.70 min for 1.8-mls articaine and 56.25 min for 1.8-mls lidocaine,^40^ and the other for maxillary incisor labial infiltrations, with 24.5 min for 0.6-mls articaine and 23.8 min for 0.6-mls lidocaine.^41^

#### LA-related adverse effects

As with previous systematic reviews, this systematic review found no incidence of permanent paraesthesia in any of the studies, which included follow up for adverse effects. Neither reviews nor individual studies specify a standard definition of ‘paraesthesia’.

### Overview of previous systematic reviews

The broader systematic reviews of articaine all recognise articaine’s equal or superior efficacy when compared with lidocaine for routine dental treatment.^10,43,44^ Katyal^10^ found articaine superior to lidocaine in posterior first molar anaesthesia.^10^ Out of 1022 study participants, Brandt et al.^43^ found articaine superior to lidocaine for all dental infiltrations and for mandibular block anaesthesia in healthy teeth.^43^ Soysa et al.^44^ found articaine superior to lidocaine for all mandibular interventions.^44^ None of the reviews reported any short or long-term paraesthesia.

The most recent systematic review by Soysa et al. in 2019 reviewed RCTs of articaine from 2000 to 2018.^44^ Soysa et al. included eighteen studies for meta-analysis. Twelve of these studies were excluded by this systematic review because one involved non-routine third molar extraction and one involved the non-standard Gow-Gates block technique. The remaining eight studies were assessed by us as having high risk of bias due to lack of description of the allocation or randomisation process, lack of blinding by the person administering the anaesthetic and anaesthetic cartridges not being masked. The meta-analysis in this review included three studies not included in Soysa’s. These were: Haase et al.^34^, Kumar et al.^40^ and Srisurang et al.^42^. All were RCTs assessed as low-to-medium risk of bias using the Cochrane Risk of Bias 2 guidelines.

Other comparisons of this meta-analysis to Soysa et al.’s review^44^ are listed below:

– Both reviews had the same outcome measure—anaesthetic success of articaine compared to lidocaine; however, Soysa et al. only included studies, which measured the efficacy of an agent without requiring re-anaesthesia, whereas this review included studies of supplementary anaesthesia techniques.
– Soysa et al. analysed studies involving posterior teeth only, whereas this review included studies of all teeth.
– Soysa et al. excluded studies using <0.9 mL of anaesthetic solution, whereas this review included all RCTs comparing articaine and lidocaine regardless of anaesthetic amount.
– Soysa et al. included studies involving the Gow-Gates block anaesthesia technique whereas this review only included studies of routine dental infiltrations and the traditional inferior alveolar nerve blocks.

The overall results from this review also differed slightly from the conclusions made by Soysa et al. Both reviews found that articaine is more likely to achieve anaesthetic success than lidocaine in combined analysis, mandibular infiltration and block anaesthesia. This review found that this conclusion was also true for maxillary infiltrations, unlike Soysa et al. who found no significant difference in anaesthetic success between articaine and lidocaine for maxillary infiltrations. Both reviews note the potential effect of medium-to-high rates of heterogeneity on the review outcomes.^44^

Paxton and Thome^8^ and Yapp et al.^11^ conducted literature reviews of articaine both recognising a general trend of articaine outperforming lidocaine in anaesthetic efficacy.^8,11^ Yapp et al. stated that articaine is a safe and effective LA for all routine dental procedures for patients of all ages, and that no conclusive evidence demonstrates articaine neurotoxicity over any other dental anaesthetic.^11^

Reviews comparing articaine efficacy to that of lidocaine’s in patients with irreversible pulpitis found that both LAs lack efficacy for mandibular block anaesthesia, but that articaine’s rate of anaesthetic success was significantly superior to lidocaine for supplementary mandibular infiltrations following failed mandibular block anaesthesia to anaesthetise symptomatic teeth.^45–47^ In general, these reviews found articaine superior to lidocaine in achieving anaesthetic success and for pain control in symptomatic teeth.^45–47^

Successful anaesthesia rates for mandibular block anaesthesia in healthy versus inflamed pulps are ~70% compared to 30%. Teeth with irreversible pulpitis are more difficult to anaesthetise compared to asymptomatic teeth.^11,23–26^

### General overview of previous articaine studies

#### Anaesthetic efficacy

For mandibular block anaesthesia efficacy in teeth with irreversible pulpitis undergoing endodontic treatment, articaine has an anaesthesia success rate of 87% compared to 60% with lidocaine.^48^ For anaesthesia of mandibular teeth following failed mandibular block anaesthesia, intraosseous anaesthesia with articaine has a success rate of ~86% in mandibular posterior teeth,^49^ and supplementary articaine mandibular buccal infiltrations have a success rate of ~42–73%.^50–52^

For mandibular incisors, combined articaine labial and lingual infiltrations provide effective pulpal anaesthesia compared to labial alone with anaesthetic duration <60 min.^53^ However, higher than normal doses of buccal infiltrations of articaine can effectively anaesthetise maxillary teeth for extractions without a need for palatal anaesthesia.^54,55^

Most studies and reviews did not find a significant difference in anaesthesia success comparing articaine buccal infiltration with mandibular block anaesthesia in adults or children, recognising that articaine buccal infiltrations can be used as a substitute for lidocaine mandibular block anaesthesia, especially for paedodontic pulpal treatments.^10,56–64^ An outlier study by Arrow in 2012 found that mandibular block anaesthesia of both articaine and lidocaine had higher anaesthetic success than buccal infiltrations of both anaesthetics alone.^65^

For mental/incisive nerve blocks, Batista da Silva et al.^66^ found that articaine has a higher success rate than lidocaine for anaesthetising mandibular anterior teeth, but that anaesthesia could only be considered successful for premolars, not anterior teeth.^66^

#### Anaesthetic concentrations

A comparison of mandibular block anaesthesia with 2 and 4% articaine for extraction of mandibular posterior teeth acknowledges that both concentrations give adequate anaesthesia with no significant difference, except that 2% articaine results in shorter soft tissue anaesthesia.^67^ Two percent articaine maybe advantageous for children due to its lower maximum serum concentration and shorter serum half-life.^17^

#### Vasoconstrictor concentrations

Articaine provides more efficacious anaesthesia when combined with adrenaline than without,^4,8,14,68,69^ with no significant difference between the 1:100,000 and 1:200,000 concentrations of the vasoconstrictor.^70^ Kammerer et al. stated in 2012 that although articaine with 1:100,000 vasoconstrictor had a faster onset than that with no vasoconstrictor, both provide adequate anaesthesia when administered as mandibular block anaesthesia for mandibular extractions.^71^ However, in a subsequent 2014 study, the same researcher recognised that articaine with no vasoconstrictor had a much shorter anaesthetic effect and that LAs with vasoconstrictor produce longer, deeper anaesthesia.^72^

The majority of studies comparing different adrenaline concentrations of 4% articaine found no significant difference in pulpal anaesthesia success rates between 1:100,000 and 1:200,000 concentrations; however, the 1:100,000 adrenaline may have an insignificant advantage over the 1:200,000^4,8,68,73–75^ and may be more efficacious than the 1:200,000 adrenaline for extractions of maxillary third molars.^76^

#### Anaesthetic dose

For anaesthesia of mandibular first molars, 3.6 mls of articaine as a buccal infiltration provides more effective anaesthesia than 1.8 mls, with ~70% success rate,^77,78^ but as a supplementary anaesthetic to failed mandibular block anaesthesia, there is no difference in anaesthetic efficacy between the two doses.^79,80^ In the maxilla, a dose of 1.2 mls of articaine as a buccal infiltration is more efficacious than a dose of 0.6–0.9 mls,^81^ meaning a higher dose results in a higher rate of anaesthetic success.

#### Anaesthesia in children

The safety of articaine use in children under 4 years of age was documented in a 1989 retrospective report by Wright et al. reviewing 211 paedodontic cases using articaine. No adverse reactions were observed, therefore, the review stated that articaine is safe to use in children under age 4.^82^ Articaine was recognised as safe and efficacious in children of all ages in a 2011 comprehensive review of articaine.^11^ A subsequent 2018 study found that there is no difference between articaine and lidocaine in frequencies of anaesthetic-related adverse events in children.^83^

#### Adverse effects

Paraesthesia associated with dental anaesthesia is defined as numbness or tingling of the mouth and face.^12^ The hypothesized association of articaine having an increased risk of paraesthesia following mandibular block anaesthesia may have been precipitated with Haas and Lennon’s^84^ retrospective study of reported paraesthesia cases in Ontario’s Professional Liability Program between 1973 and 1993. The study associated articaine with more cases than other LAs by comparing the number of LA cartridges used in relationship to market share of the type of LA.^84^

Follow-up retrospective studies conducted by Gaffen and Haas in 2009, again reviewed the same database from 1999 to 2008, reporting that the incidence of non-surgical paraesthesia during the studied time frame was 1 in 609,000. The same study stated that prospective studies of anaesthesia-related adverse events are challenging to undertake due to difficulty getting ethics approval for a cohort large enough to detect any statistical significance as LA-related paraesthesia occurrence is rare.^85^ A subsequent 2010 review involving a researcher from the previous two mentioned studies reported that the incidence of adverse effects from articaine was ~1 in 4,159,848 and that 4% LA solutions had the highest incidence of adverse reported events based upon dental LA market share data.^86^

Other systematic reviews and RCTs have not been able to find any scientific evidence corroborating the hypothesis that articaine is associated with increased risk of permanent paraesthesia.^6,11,87^ Three studies in 1995,^88^ 2000^89^ and 2007^90^ involving the same researcher revealed equal distributions of nerve damage among anaesthetic solutions, with lidocaine having more associations with LA-related adverse events than articaine. A 2001 study involving 882 articaine interventions revealed no incidences of temporary or permanent nerve damage.^6^

Yapp et al.’s^11^ comprehensive literature review of articaine could not find any scientific evidence supporting articaine’s association with increased paraesthesia, stating that LA-related paraesthesia is uncommon, with the incidence was found to be between 1 in 726,000 and 1 in 785,000.^11^ The review listed direct needle trauma, intra-neural haematoma formation, fascicular pattern and LA toxicity as the potential explanation for LA-related nerve involvement. Yapp et al.’s review also judged previous retrospective studies from Haas and Lennon^84^, Hillerup and Jensen^91,92^, Gaffen and Haas^85^, Garisto et al.^86^ associating articaine with higher incidence of paraesthesia to be of low-level evidence, biased in data recruitment, and not robust enough in protocol to derive any clinical recommendations.^11^

Toma et al.’s comprehensive 2016 synopsis of studies on dental anaesthetic-related adverse events also could not find any scientific evidence corroborating claims of articaine’s association with adverse events. The review stated that the evidence for anaesthetic-related neurotoxicity is lacking and reached the same conclusion as Yapp et al. in 2011, that the reports and studies suggesting that articaine is associated with higher frequency of neurotoxicity are of poor quality and at high risk of bias.^87^

A 2015, in vitro study of anaesthetic effect on human neuroblastoma cells reported that with increasing concentrations, all anaesthetics eventually resulted in induced cell death, but articaine and ropivacaine were the least neurotoxic; mepivacaine, prilocaine and lidocaine were considered of medium neurotoxicity, and bupivacaine resulted in the most rapid nerve cell death.^93^

Another in vitro study of anaesthetic effect on rodent neural cells found that articaine resulted in the most effective blocking of nerve action potentials compared to lidocaine and mepivacaine.^13^

### Limitations

This systematic review and meta-analysis were limited to English resources and excluded studies involving non-routine dental treatment and anaesthesia techniques, for example, third molar surgery and digital anaesthesia. In addition, the studies included for meta-analysis had a medium-to-high level of heterogeneity. These factors could have affected the outcomes of the meta-analysis.

### Discussion of updated search results from February 2020 to May 2021

The authors conducted an updated search to find studies released between February 2020 and May 2021 that were not available or published at the time of the initial research. The purpose of the exercise was to assess the potential impact of the data of new RCTs on the current study outcomes.^94^ The search discovered nine reviews and ten studies.

Five reviews involved third molar extraction surgeries. Three involved complex surgical extractions and were excluded, and the remaining two systematic reviews with meta-analysis revealed data relevant to this review. The first studied the safety and efficacy of 4% articaine in mandibular third molar extractions finding that 4% articaine is a safe choice for third molar extractions requiring less supplemental anaesthesia, with a shorter onset time than the other amide LAs.^95^ The second study analysed articaine and hypesthesia in third molar extractions concluding that the use of articaine during third molar extraction does not increase the risk of hypesthesia compared to other LAs.^96^

Two reviews involved paediatric dentistry. The first analysed specialist views on articaine administration for children and concluded that articaine use for paediatric dentistry is common but supported by limited evidence.^97^ The second compared studies of articaine and lidocaine for dental procedures in paediatric patients finding that articaine is more effective than lidocaine, but the margin of difference in their study was small.^98^

Eleven new studies were assessed for potential inclusion in future meta-analysis. Seven were excluded due to: not being RCTs, not comparing articaine and lidocaine, only using articaine with no comparison LA, not using a reliable measure of intervention, inadequate blinding and studies involving complex, surgical third molar extractions.

Four studies should be assessed for inclusion in a subsequent meta-analysis comparing articaine and lidocaine for routine dental procedures. The conclusion of these studies is:

- Articaine showed faster onset and duration of anaesthesia than lidocaine for buccal infiltrations.^99^
- Articaine is an efficient and safe LA to treat children between ages three and four.^100^
- Articaine’s anaesthetic success rate was significantly higher than lidocaine’s and mepivacaine’s for supplemental buccal infiltrations.^101^
- Articaine can be used as buccal infiltration for invasive treatment of mandibular molars in children ages eight to fifteen. There was no difference in anaesthesia success between lidocaine mandibular blocks and an articaine buccal infiltrations in this study.^64^

The conclusions from the latest RCTs that were not available at the time of our meta-analysis aligned with our included studies. The corroboration of these newer studies give reassurance that our meta-analysis results are relevant to the present day.

## Conclusion

The conclusion of this systematic review supports that articaine is a safe and efficacious LA for all routine dental procedures in patients of all ages. The meta-analysis found articaine more likely to achieve successful anaesthesia than lidocaine in maxillary and mandibular infiltration anaesthesia, and mandibular block anaesthesia for asymptomatic and symptomatic teeth. Neither anaesthetic has a higher association with anaesthetic-related adverse effects.
