# Correction: Articaine in dentistry: an overview of the evidence and meta-analysis of the latest randomised controlled trials on articaine safety and efficacy compared to lidocaine for routine dental treatment

**DOI:** 10.1038/s41405-021-00085-2

**Published:** 2021-08-11

**Authors:** Erica Martin, Alan Nimmo, Andrew Lee, Ernest Jennings

**Affiliations:** 1 General Dentistry, College of Medicine and Dentistry, James Cook University, Cairns, QLD Australia; 2 Medical Science, College of Medicine and Dentistry, James Cook University, Cairns, QLD Australia; 3 Preventative Dentistry and Indigenous Oral Health, College of Medicine and Dentistry, James Cook University, Cairns, QLD Australia; 4 Anatomy, College of Medicine and Dentistry, James Cook University, Cairns, QLD Australia

**Keywords:** Dental anaesthesia, Dentistry

Correction to: *BDJ Open* 10.1038/s41405-021-00082-5, published online 17 July 2021

When originally published on 17 July 2021, there was an incorrect value in the ‘Articaine safety’ section: ‘Malamed et al.’s 2001’s multi-centre trial involving the comparison of 2% lidocaine with 4% articaine on 1325 patients aged 4–8 years of age found that articaine was well-tolerated and safe for use in routine clinical dentistry’ should have read ‘Malamed et al.’s 2001’s multi-centre trial involving the comparison of 2% lidocaine with 4% articaine on 1325 patients aged 4–80 years of age found that articaine was well-tolerated and safe for use in routine clinical dentistry.’

The authors apologise for any inconvenience caused.

